# The Use of TNF-α Inhibitors in Active Ankylosing Spondylitis Treatment

**DOI:** 10.7759/cureus.61500

**Published:** 2024-06-01

**Authors:** Georgios Zouris, Dimitrios Stergios Evangelopoulos, Ioannis S Benetos, John Vlamis

**Affiliations:** 1 5th Orthopaedic Department, General Hospital "Asklepieio" Voulas, Athens, GRC; 2 Postgraduate Training Program, KAT Hospital, National and Kapodistrian University of Athens School of Medicine, Athens, GRC; 3 3rd Orthopaedic Department, KAT Hospital, National and Kapodistrian University of Athens School of Medicine, Athens, GRC; 4 Metabolic Bone Diseases Department, KAT Hospital, National and Kapodistrian University of Athens School of Medicine, Athens, GRC

**Keywords:** ankylosing spondylitis, adalimumab, certolizumab pegol, infliximab, golimumab, etanercept, anti tnf-alpha, tnf-a inhibitors, anti tnf-a, tnf alpha inhibitors

## Abstract

Ankylosing spondylitis (AS) is a challenging disease, characterized by chronic inflammation and structural damage primarily affecting the axial skeleton, while extra-articular manifestations may also appear. This results in the deterioration of patients’ quality of life. Over the past few decades, tumor necrosis factor-α (TNF-α) inhibitors have revolutionized the management of AS, offering substantial relief from symptoms and improving patient outcomes. The aim of this review is to assess the efficacy of TNF-α inhibitors in patients with active AS.

A search was performed in the PubMed database using the following keywords: ("TNF alpha inhibitors" OR "anti TNF-a" OR "TNF-a inhibitors" OR "anti TNF-alpha" OR "Etanercept " OR "Golimumab" OR "Infliximab" OR "Certolizumab pegol" OR "Adalimumab") AND "ankylosing spondylitis". The search was completed in February 2024, and 35 studies were included in this review following PRISMA guidelines. The findings reveal evidence supporting the efficacy of TNF-α inhibitors in reducing inflammation, preventing structural damage, and enhancing overall well-being in AS patients. Overall, TNF-α inhibitors have emerged as a cornerstone in the therapeutic algorithm against AS with a very satisfactory safety profile.

## Introduction and background

Ankylosing spondylitis (AS) is a chronic, inflammatory condition affecting the axial skeleton, showcasing a diverse array of clinical manifestations. While primarily targeting the spine, it also involves the sacroiliac joints and peripheral joints. The main features of the disease include persistent spinal pain, progressive stiffness, and resultant postural abnormalities. Additionally, AS may exhibit extra-articular symptoms such as inflammatory bowel disease (observed in up to 50% of patients), uveitis (affecting 25%-35% of patients), and psoriasis (present in roughly 10% of patients). Furthermore, AS poses an elevated risk of cardiovascular ailments due to systemic inflammation, as well as pulmonary complications stemming from restricted rib cage and spinal mobility. Patients with AS are also susceptible to vertebral fractures, atlanto-axial joint subluxation, spinal cord injuries, and occasionally, cauda equina syndrome [1].

AS occurs mainly in people under the age of 40, with about 80% of the cases diagnosed before the age of 30, and less than 5% of the cases diagnosed after the age of 45. The disease is more common in men than in women, with a ratio of about 2-3:1, and relatives of AS patients have an increased likelihood of developing the disease compared to the general population [1-3].

The main features of the disease are enthesitis and arthritis with early involvement of the sacroiliac joints and the joints of the lumbar spine. This inflammation can cause progressive ankylosis of these joints. Other large joints are also involved such as the shoulder, knee (25%-50%), and hip (20%). Symptoms usually begin with progressive pain in the back and lower back. As the disease progresses, the pain may wake the patient in the morning and be accompanied by morning stiffness that subsides with exercise. Also due to enthesitis, they can experience chest pain, epicondylitis, Achilles tendonitis, and plantar fasciitis [1,2,4].

The main therapeutic goals include the reduction of pain and spinal stiffness to improve the patient’s quality of life. Non-drug treatments should include consistent physical activity, posture coaching, and rehabilitation exercises [1,2]. The available medication choices for AS include non-steroidal anti-inflammatory drugs, biological treatment (tumor necrosis factor-α [TNF-α] inhibitors, monoclonal antibodies), JAK inhibitors, corticosteroids, and disease-modifying anti-rheumatic drugs [2,5].

The goal of this review is to focus on TNF-α inhibitors and evaluate their use and efficacy on active AS patients. TNF-α is a potent cytokine that induces inflammatory processes crucial to the pathogenesis of many inflammatory and autoimmune diseases including AS [6].

There are five TNF-α inhibitors that can be used for the treatment of AS: (a) infliximab (IFX), a chimeric (75% human/25% mouse) monoclonal antibody that impedes the cytokine's ability to activate the cellular TNF receptor complex. It is administered intravenously (IV). (b) Adalimumab (ADA), a completely humanized monoclonal antibody composed of recombinant immunoglobulin (IgG1) targeting TNF-α, with the added capability of inhibiting the attachment of TNF-α to its receptor sites. It is administered subcutaneously (SC). (c) Etanercept (ETN), a dimeric chimeric protein genetically engineered by fusing the extracellular binding domain of human TNF receptor-2 to the Fc domain of human IgG1. It prevents the binding of the TNF to its receptors on the cell surface. It is administered SC. (d) Golimumab (GLM), a fully human monoclonal antibody that has the ability to attach to both soluble and transmembrane TNFs, effectively blocking their interaction with TNF receptors and halting TNF activity. It is administered IV or SC. (e) Certolizumab pegol (CZP), a humanized antigen-binding fragment (Fab’) of a monoclonal antibody linked to polyethylene glycol. It has been shown to neutralize soluble, membrane-bound, human TNFα. It is administered SC [7].

**Figure 1:**
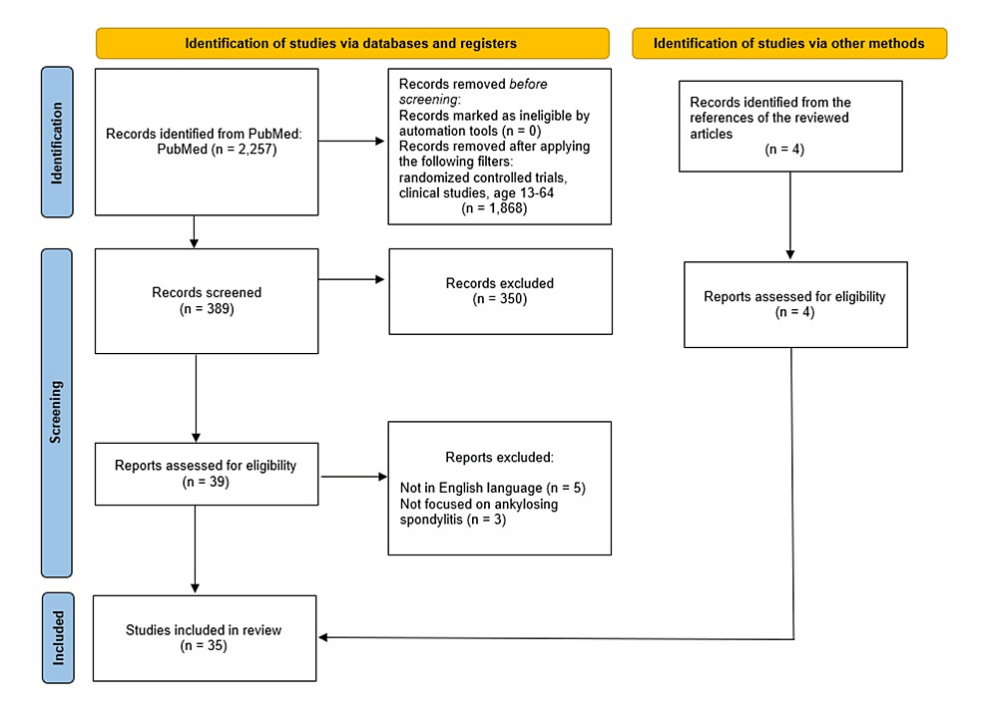
The flowchart of the present literature review

As mentioned before, the purpose of this literature review is to summarize the use and effectiveness of TNF-α inhibitors as a treatment in AS patients and assess whether patients’ quality of life improves and if the adverse events are serious enough to avoid using them. The review was performed following PRISMA guidelines on the online PubMed database. Inclusion criteria were randomized controlled trials and clinical studies published after 2000, the participants could be humans of any gender aged 13 to 64 years. Also, the studies should be in English language. The keywords used for the search were “TNF alpha inhibitors”, “anti TNF-a”, “TNF-a inhibitors”, “anti TNF-alpha”, “Etanercept”, “Golimumab”, “Infliximab”, “Certolizumab pegol”, “Adalimumab”, and “ankylosing spondylitis”. The primary search included 2257 articles. By excluding articles that didn’t match our criteria, 389 studies remained. After evaluating titles and abstracts, 39 articles remained, and after the final evaluation, 35 studies were included in this review. Each of these studies investigates the effectiveness of one TNF-α inhibitor. We included eight studies for IFX, 10 for ADA, nine for ETN, five for GLM, and three for CZP. The process is summarized in Figure 1.

## Review

Evaluation criteria

Several criteria can be used to assess the improvement of AS patients under treatment:

Criteria That Assess the Disease Activity

(a) Bath AS Disease Activity Index (BASDAI) includes six questions: fatigue, spinal pain, peripheral joints, entheses, the intensity of morning stiffness, and duration of morning stiffness. Each question is scored on a numeric rating scale (NRS) or a 10 cm visual analog scale (VAS), and the final BASDAI score is calculated by summing the first four questions and the average of the last two, and dividing the result by five. The score ranges from 0 (no disease activity) to 10 (very active disease). A score ≥4 defines active disease [8].

(b) The Ankylosing Spondylitis Disease Activity Score (ASDAS) includes five questions: back pain, peripheral pain/swelling, duration of morning stiffness, and patient global assessment of disease activity, with either the erythrocyte sedimentation rate (ASDAS-ESR) or the C-reactive protein (ASDAS-CRP). ASDAS score ≤1.3 is low disease activity, between 1.3 and 2.1 is moderate, between 2.1 and 3.5 is high, and ≥3.5 is very high disease activity [8].

Criteria That Assess Treatment Response

(a) ASsessment in AS (ASAS) International Working Group criteria include four domains: patient global, pain, function (assessed by BASFI), and inflammation (BASDAI questions 5 and 6). To achieve an ASAS20 response, improvement of at least 20% is required in three out of four domains, with a minimum increase of one unit on a scale ranging from 0 to 10. In the fourth domain, there must be no deterioration exceeding 20%, and a minimum of one unit on a 0-10 scale. For ASAS40 response, improvement of at least 40% is required in three out of four domains with a minimum increase of two units on a scale ranging from 0 to 10. In the fourth domain, there must be no deterioration exceeding 20%, and a minimum of one unit on a 0-10 scale. ASAS 5/6 response evaluates the previous four domains plus two: CRP and spinal mobility. To achieve this, at least 20% improvement is required in at least five out of six domains. ASAS partial remission defines a very low disease activity. Achieving a value of 2 or less (on a scale of 0-10) is required in all four domains [8].

(b) BASDAI 50 response is the improvement of at least 50% in the BASDAI score or an absolute increase of 2 units (on a scale of 0-10) after three months of treatment [8].

Criteria for Spinal Mobility

Bath AS Metrology Index (BASMI) evaluates five measures: tragus-to-wall distance, modified Schober’s test, cervical rotation, lateral spinal flexion, and intermalleolar distance. These five measurements get a score on a scale of 0-2. Summing them, we get a final score on a scale of 0-10 (0 no mobility limitation, 10 severe mobility limitation) [8].

Criteria for Physical Function

Bath AS Functional Index (BASFI) includes 10 questions: eight focusing on functional anatomy and two on the capacity to manage daily activities. Responses are recorded on NRS or a 10 cm VAS, with "easy" and "impossible" as endpoints. The overall BASFI score is calculated as the average of the 10 questions and falls within a range of 0-10 [8].

Quality of Life

(a) Short Form-36 (SF-36) is a questionnaire of 36 questions that evaluates the health status. It includes an eight-domain score (physical functioning, role physical, bodily pain, general health, vitality, social functioning, role emotional, and mental health), which ranges from 0 to 100 (higher scores indicate a better health status), a physical summary score (SF-36 PCS), and a mental summary score (SF-36 MCS) calculated by specific algorithms [9].

(b) The Ankylosing Spondylitis Quality of Life Questionnaire (ASQoL) includes 18 items that assess the impact of AS in the patient’s life. Its score ranges from 0 to 18, and lower scores reflect a better quality of life [10].

Drug assessment

Infliximab

In 2010, Inman and Maksymowych [11] published the results of low-dose IFX in patients with AS. From a total of 76 patients, 39 received IV IFX 3 mg/kg and 37 received a placebo at weeks 0, 6, and 12. The primary endpoint was an ASAS20 response at week 12. ASAS20 was achieved by 53.8% of patients in the IFX group compared to 30.6% of patients in the placebo group (p=0.042). ASAS40 response was achieved by 46.2% of IFX patients, compared to 8.3% of placebo patients (p<0.001). ASAS 5/6 response was achieved by 51.3% of IFX patients, compared to 2.8% of placebo patients (p<0.001). Also, at week 12, patients receiving IFX had a greater change in BASDAI (-2.1 compared to -0.7 in placebo patients) (p=0.003) and BASDAI 50 was achieved by 28.2% of IFX patients, compared to 11.1% of placebo patients (p=0.064). After week 12, the open-label phase of the study started and all patients received IFX till week 50. By week 50, there were no significant differences between the two treatment groups in ASAS and BASDAI responses and reported improvements in every domain of the SF-36 questionnaire. The results of this study suggest that 3 mg/kg IFX IV reduces the symptoms of AS and improves the patients’ health status. Only two patients withdrew from the study because of adverse events, suggesting that IFX was generally a safe and well-tolerated treatment [11].

In 2005, Van Der Heijde et al. [12] presented the results of a trial, which also suggested that IFX was effective and safe for AS patients. From a total of 279 patients, 201 were randomly assigned to receive 5 mg/kg IFX IV and 78 to receive a placebo, at weeks 0, 2, 6, 8, 12, and 18. At week 24, the IFX group contained 61.2% ASAS20 responders, as compared with 19.2% of the placebo group (p<0.001). ASAS40 was achieved by 47% of patients in the IFX group but only by 12% of patients in the placebo group (p<0.001). ASAS 5/6 was achieved by 49% of IFX patients compared with 8% of placebo patients (p<0.001) and ASAS partial remission was achieved by 22.4% of IFX patients, but only by 1.3% of placebo patients (p<0.001). Disease activity was scientifically reduced in the IFX group patients, as 51% of them had at least a 50% improvement in their BASDAI score, compared with only 10.7% of placebo patients achieving that improvement (p<0.001). Spinal mobility also improved in the IFX group patients, as they showed a greater improvement in all measures of the BASMI score except for the lumbar flexion element (Schober test). A notable improvement in chest expansion and CRP drop was observed. There was an improvement in the physical component score of SF-36, but there were no differences in the mental component. Serious adverse events were experienced only by 3.5% of patients in the IFX group and 2.7% in the placebo group [12].

Van Der Heijde et al. [13] based on the previous study also assessed the outcome of IFX therapy in patient productivity at work and published the results in 2006. At week 24, the percentage of patients in the IFX group reporting that their physical health affected their daily activities or work was reduced from 94% to 64% (p<0.001), as compared with the placebo group which reported reductions from 97% to 87% (p<0.05). The percentage of patients reporting emotional impact also decreased from 54% to 36% in the IFX group (p<0.001), as compared with a nonsignificant change from 55% to 52% in the placebo group (p>0.05). Also, at week 24 of therapy, the productivity score increased with a median percent change of 62% in the IFX group, contrasted with just an 11% change in the placebo group [13].

Breban et al. [14] examined 50 patients who received three infusions of IFX (5 mg/kg) at weeks 0, 6, and 12 in a 24-week open-label study. The results were more than encouraging: 98% of patients had at least a 20% improvement in the global assessment of pain (GAP), 94% were ASAS20 responders, and 70% had partial remission. Forty patients (80%) experienced adverse events. All of them were mild except for two serious adverse events, both unrelated to the treatment [14].

Temekonidis et al. [15] examined 25 patients in a 12-month open-label study. The patients received IFX 5 mg/kg IV at weeks 0, 2, and 6 and then every eight weeks. Twenty-three patients (92%) had a GAP reduction of more than 20%, 21 patients (84%) had a reduction of 50%, and 13 patients (52%) had a reduction of 70%. Fifteen patients (60%) were BASDAI 50 responders, 22 patients (88%) achieved an ASAS20 response, and 18 patients (72%) had partial remission. They also reported a significant reduction in CRP and ESR [15].

Braun et al. [16] carried out a 12-week multicenter randomized placebo-controlled trial that showed that IFX is effective for AS patients. In this study, 35 patients had IV IFX (5 mg/kg) at weeks 0, 2, and 6, and 35 patients had placebo at the same intervals. Fifty-three percent of the IFX group patients achieved BASDAI 50 response in week 12, in contrast with the placebo group, with 9% achievers of BASDAI 50. Patients in the IFX group achieved a significant improvement in function (BASFI score reduced from 5.5 to 3.4) (p<0.0001) in contrast with the placebo group patients that did not improve significantly (BASFI score from 5.1 to 5.0) (p=0.54). Serum CRP and ESR levels were significantly decreased in the IFX group at week 12 (p<0.0001), while there was no decrease in the placebo group. While IFX was well tolerated in general, three patients had to stop their treatment due to serious adverse events. One patient developed systemic tuberculosis in the lymph nodes and spleen. Another developed high fever with enlarged lymph nodes and pulmonary lesions, and the third had transient leucopenia [16].

Braun et al. [17] examined the long-term efficacy of IFX by extending the previous study in an open-label observational study. Fifty-four patients received IFX (5 mg/kg) every six weeks till week 54. At 54 weeks, 47% of IFX/IFX group patients achieved BASDAI 50 response and 18% of them achieved partial remission. In the placebo/IFX group, 50% of patients had a BASDAI 50 response and 17% of them achieved partial remission [17]. Forty-nine patients continued to receive IFX every six weeks till week 156. The results of this three-year study suggest that patients who received IFX for a long time sustained a good clinical outcome without losing its efficacy, and had good tolerance to it [18].

Summarizing the findings of all IFX studies (Table 1), we can note the following important points:

Effectiveness: Across all studies, IFX demonstrated significant effectiveness in improving AS symptoms and reducing disease activity, as measured by various endpoints (ASAS20, ASAS40, BASDAI, BASFI). Higher doses (5 mg/kg) generally showed more robust results compared to lower doses (3 mg/kg).

Safety: IFX was generally well-tolerated with mild to moderate adverse events. A small percentage of patients experienced serious adverse events, including infections.

Long-term outcomes: Sustained efficacy over extended treatment periods was observed, indicating IFX's potential for the long-term management of AS.

Functional and quality-of-life improvements: Significant improvements were noted in physical health, spinal mobility, productivity at work, and quality-of-life metrics (SF-36 scores), demonstrating IFX's broad impact on daily functioning and well-being.

**Table 1:** Summary of studies for infliximab IFX, infliximab; IV, intravenously; ASAS, ASsessment in AS; AS, ankylosing spondylitis; BASDAI, Bath AS Disease Activity Index; BASFI, Bath AS Functional Index; GAP, global assessment of pain; CRP, C-reactive protein; ESR, erythrocyte sedimentation rate.

Study	Patient groups/Dose	Frequency	Results
Inman and Maksymowych, 2010 [11]	(a) IFX (3 mg/kg) IV; (b) placebo	Weeks 0, 6, 12	Week 12 (a) vs (b):
			ASAS 20: 53,8% vs 30.6%, ASAS 40: 46.2% vs 8.3%, ASAS 5/6: 51.3% vs 2.8%
			BASDAI 50: 28.2% vs 11.1%
Van Der Heijde et al., 2005 [12]	(a) IFX (5 mg/kg) IV, (b) placebo	Weeks 0, 2, 6, 8, 12, 18	Week 24 (a) vs (b):
			ASAS 20: 61.2% vs 19.2%, ASAS 40: 47% vs 12%, ASAS 5/6: 49% vs 8%, ASAS partial remission: 22.4% vs 1.3%
			Improvement in BASDAI and BASFI in group (a)
Van Der Heijde et al., 2006 [13]	The same patient sample and drug dose with the previous study	Weeks 0, 2, 6, 8, 12, 18	Physical health affecting activities/work:
			Group (a): 94% → 64%, group (b): 97% → 87%
			Mental/emotional health affecting activities/work:
			Group (a): 54% → 36%, group (b): 55% → 52%
			Productivity score:
			Group (a): ↑ 62%, group (b): ↑ 11%
Μ. Breban et al., 2002 [14]	IFX (5 mg/kg) IV	Weeks 0, 6, 12	20% GAP improvement: 98%
			ASAS 20: 94%, ASAS partial remission: 70%
Temekonidis et al., 2003 [15]	IFX (5 mg/kg) IV	Weeks 0, 6, 12 and then every 8 weeks up to 12 months	>20% GAP improvement: 92%, 50% GAP improvement: 84%, 70% GAP improvement: 52%
			BASDAI 50: 60%
			ASAS 20: 88%, ASAS partial remission: 72%
Braun et al., 2002 [16]	(a) IFX (5 mg/kg) IV, (b) placebo	Weeks 0, 2, 6	Week 12 (a) vs (b):
			BASDAI 50: 53% vs 9%
			BASFI: (a) 5.5 → 3.4, (b) 5.1 → 5.0
			↓ CRP ↓ ESR
Braun et al., 2003 [17]; Braun et al., 2005 [18]	Extension of the previous study up to 3 years. (a) IFX/IFX, (b) placebo/IFX	Every 6 weeks up to 3 years	Week 54 (a) vs (b):
			BASDAI 50: 47% vs 50%
			ASAS partial remission: 18% vs 17%
			Results remained satisfactory up to 3 years of treatment

Adalimumab

Van Der Heijde et al. [19] assessed the efficacy and safety of ADA in a multicenter, randomized, double-blind, placebo-controlled trial. In this study, out of 315 patients, 208 received 40 mg ADA SC every other week (eow), and 107 of them received a placebo for 24 weeks. At week 12, ASAS20 response was reported by 58.2% of ADA patients compared with 20.6% of placebo patients (p<0.001), while BASDAI 50 response was reported by 45.2% and 15.9%, respectively (p<0.001). At week 24, 44.7% of ADA patients achieved an ASAS 5/6 response, compared with 12.1% of placebo patients (p<0.001), ASAS40 was achieved by 39.4% and 13.1% of patients, respectively, and ASAS partial remission was achieved by 22.1% and 5.6%, respectively (p<0.001). It is worth mentioning that after week 12, 74 patients who received a placebo before switched over to treatment with ADA and became ASAS20 responders at week 16. In contrast to administering a placebo, treatment with ADA suggested statistically significant improvements in all signs and symptoms of AS, like CRP, spinal mobility (BASMI), and enthesitis. Seventy-five percent of ADA patients experienced adverse events compared with 59.8% of placebo patients. However, most of them were mild and medically manageable. Serious adverse events were reported by 2.9% of ADA patients, but only one patient who developed hypersensitivity to ADA withdrew from the study. ADA, overall, was well tolerated [19].

The impact of ADA on patients’ quality of life was also measured by Davis et al. [20] in the same patient sample of the previous study. A statistically significant improvement was observed in SF-36 PCS and ASQoL scores in ADA patients, compared to placebo patients, but no difference was observed in SF-36 MCS scores. At week 12, the ASQoL score was reduced by a bigger margin (-3.2±0.3) in ADA patients than the more modest (-1.0±0.4) in placebo patients. These satisfying improvements were maintained until week 24 [20].

Van Der Heijde et al. [21] also evaluated the long-term efficacy of ADA, expanding the study as an open-label, for up to five years, and the results were encouraging. Two hundred and sixty-one patients continued for up to two years, 236 patients up to three years, and 202 patients completed the study up to five years. The five-year analysis included only 125 patients who received ADA from the first week of the initial study. Also, 72 patients who were ASAS20 non-responders started to receive 40 mg ADA weekly. Treatment response (ASAS), disease activity (BASDAI), physical function (BASFI), spinal mobility (BASMI), and quality of life, all maintained their improvement. At year 2, 64.5% (200/310) of patients were ASAS20 responders, 50.6% were ASAS40 responders, 58.9% were ASAS 5/6 responders, and 33.5% had partial remission [21]. At week 156, the BASDAI score was reduced (-3.9±3.39), so was BASFI (-3.0±2.10) and ASQoL (-5.4±4.36). In year 5, all scores kept improving. BASMI score was reduced (-3.7±1.7) and CRP levels decreased from 1.30 mg/dl to 0.50 mg/dl at week 12 [22,23].

Eleven patients from the initial study who were diagnosed with total spinal ankylosis were also evaluated. Six of them were randomized to ADA and five to placebo. At week 12, none of the placebo-treated patients achieved an ASAS20 response, whereas 50% of ADA-treated patients achieved an ASAS20 response, and 33% achieved an ASAS40, ASAS 5/6, and BASDAI 50 response. Finally, all 11 patients received ADA for at least one year. At that point, 8/11 patients achieved an ASAS20 response and one achieved partial remission [24].

Maksymowych et al. [25] examined the effect of ADA on biomarkers that are predictive of structural damage in AS. They were (a) serum metalloproteinase 3 (MMP-3), an enzyme found within joint synovia and cartilage, (b) urinary type II collagen C-telopeptides (CTX-II), and (c) serum type I collagen N-telopeptides (NTX). A total of 82 patients (38 ADA 40 mg SC eow, 44 placebo) enrolled. After 24 weeks of treatment, serum concentration of MMP-3 and urinary concentration of CTX-II of ADA-treated patients had a statistically significant reduction in levels compared to placebo-treated patients, where no changes were reported. These two biomarkers are linked with AS progression and CRP levels. No changes were observed in NTX levels in either group. Also, BASDAI, BASMI, and BASFI scores had a significant improvement in the ADA group. None of the patients withdrew from the study because of adverse events [25].

Rudwaleit et al. [26] carried out a 12-week open-label study evaluating the efficacy of ADA. One thousand two hundred and fifty patients who received 40 mg ADA SC participated. At week 12, 69.9% of patients were ASAS20 responders, 53.7% were ASAS40 responders, 58% were ASAS 5/6 responders, 57.2% were BASDAI 50 responders, and 27.7% experienced partial remission [26].

Huang et al. [27] conducted a randomized, controlled trial in which 344 patients with active AS enrolled. Two hundred and twenty-nine patients received 40 mg ADA SC eow, and 115 patients received a placebo for 12 weeks followed by a 12-week open-label phase. At week 12, 67.2% of the ADA group patients achieved an ASAS20 response compared to 30.4% of the placebo group patients (p<0.001). The 32.8% of ADA group patients who did not respond initially achieved an ASAS20 response at week 24. Very high ASDAS disease category was the assessment for only 3.5% of the ADA group patients versus 40% of the placebo group patients (p<0.001) [27].

S. Kobayashi et al. [28] also assessed the effectiveness and safety of ADA in Japanese people. Two hundred and sixteen patients had a baseline BASDAI score (4.9±2.3). At week 12 and week 24 of ADA treatment, the patients had BASDAI score reductions of -1.9±2.2 and -2.0±2.6, respectively [28].

Summarizing the findings of all ADA studies (Table 2), we can note the following important points:

Effectiveness: ADA consistently demonstrates substantial efficacy in improving symptoms of AS across various measures (ASAS20, ASAS40, ASAS 5/6, BASDAI, BASMI). The efficacy is seen both in short-term (12-24 weeks) and long-term (up to five years) studies.

Quality of life: ADA significantly improves physical quality of life (SF-36 PCS, ASQoL) but has less impact on mental health (SF-36 MCS).

Biomarkers: ADA reduces levels of biomarkers (MMP-3, CTX-II) associated with structural damage in AS, indicating the potential to slow disease progression.

Safety: ADA is generally well-tolerated with a majority of adverse events being mild to moderate. Serious adverse events are relatively rare.

Long-term outcomes: Sustained improvement in disease activity, physical function, spinal mobility, and quality-of-life scores over extended periods of treatment, with efficacy maintained for up to five years.

**Table 2:** Summary of studies for adalimumab ADA, adalimumab; SC, subcutaneously; ASAS, ASsessment in AS; AS, ankylosing spondylitis; BASDAI, Bath AS Disease Activity Index; CRP, C-reactive protein; BASMI, Bath AS Metrology Index; SF-36 PCS, Short Form-36 physical component summary score; ASQoL, Ankylosing Spondylitis Quality of Life Questionnaire; BASFI, Bath AS Functional Index; MMP-3, serum metalloproteinase 3; CTX-II, urinary type II collagen C-telopeptides; NTX, serum type I collagen N-telopeptides.

Study	Patient groups/Dose	Frequency	Results
Van Der Heijde et al., 2006 [19]	(a) ADA 40 mg SC, (b) placebo	Every second week up to 24 weeks	Week 12 (a) vs (b):
			ASAS 20: 58,2% vs 20.6%
			BASDAI 50: 45.2% vs 15.9%
			Week 24 (a) vs (b):
			ASAS 40: 39.4% vs 13.1%, ASAS 5/6: 44.7% vs 12.1%, ASAS partial remission: 22.1% vs 5.6%
			(a): ↓ CRP ↓ BASMI
Davis et al., 2007 [20]	The same patient sample and drug dose with the previous study	Every second week up to 24 weeks	(a): SF-36 PCS: improvement
			ASQoL↓ (a) vs (b): (-3.2±0.3) vs (-1.0±0.4)
Van Der Heijde et al., 2009 [21,22], Van Der Heijde et al., 2015 [23]	Extension of the first as an open-label study	Every second week up to 5 years	2nd year:
			ASAS 20: 64.5%, ASAS 40: 50.6%, ASAS 5/6: 58.9%, ASAS partial remission: 33.5%
			Week 156:
			BASDAI ↓: (-3.9±3.39)
			BASFI ↓: (-3.0±2.10)
			ASQoL ↓: (-5.4±4.36)
			5th year:
			All scores continued to improve
Van Der Heijde et al., 2008 [24]	11 patients with total spinal ankylosis: (a) 6 ADA 40 mg SC, (b) 5 placebo	Every second week	Week 12 (a) vs (b):
			ASAS 20: 50% vs 0%
			ASAS 20, ASAS 40, ASAS 5/6, and BASDAI 50: 33% vs 0%
			1st year (all patients had started receiving ADA before week 24):
			ASAS 20: 8/11, ASAS partial remission: 1/11
Maksymowych, et al., 2008 [25]	(a) ADA 40 mg SC, (b) placebo	Every second week	Week 24 (a) vs (b):
			MMP-3 serum: ↓ vs unchanged
			CTX-II urinary: ↓ vs unchanged
			ΝΤΧ: unchanged in both groups
	Biomarker assessment		(a): Improvement in BASDAI, BASMI, BASFI scores
Rudwaleit et al., 2009 [26]	ADA 40 mg SC	Every second week	Week 12:
			ASAS 20: 69.6%, ASAS 40: 53.7%, ASAS 5/6: 58%, ASAS partial remission: 27.7%
			BASDAI 50: 57.2%
Huang et al., 2014 [27]	(a) ADA 40 mg SC, (b) placebo	Every second week up to 12 weeks. An open-label study followed for another 12 weeks where all patients received ADA	Week 12 (a) vs (b):
			ASAS 20: 67.2% vs 30.4%, ASAS 40: 44.5% vs 9.6%, ASAS 5/6: 55.9% vs 12.2%, ASAS partial remission: 21.8% vs 3.5%
			BASDAI 50: 49.8% vs 16.5%
			ASDAS ≥3.5: 3.5% vs 40%
Kobayashi et al., 2019 [28]	ADA 40 mg SC	Every second week	Week 12: BASDAI ↓: (-1.9±2.2)
			Week 24: BASDAI ↓: (-2.0±2.6)

Etanercept

Calin et al. [29] performed a 12-week trial that evaluated the safety and efficacy of ETN in AS patients. Forty-five patients received 25 mg ETN SC twice weekly and 39 patients received a placebo. Sixty percent of ETN patients achieved an ASAS20 response. In contrast, just 23% of placebo patients achieved an ASAS20 response (p<0.001). At week 12, nearly 50% and 25% of ETN patients were ASAS50 and ASAS70 responders, respectively, a lot more than placebo patients. Spinal mobility, pain, and the BASDAI scores also had a remarkable improvement in ETN patients. CRP and ESR had a 70% and 80% reduction, respectively. ETN was generally well tolerated, as no patient discontinued the study for safety reasons [29].

The study was followed by a 96-week open-label extension performed by Dijkmans et al. [30]. Many patients from the placebo group that switched over to ETN achieved an ASAS20 response approaching the percentage of the ETN/ETN group. Overall, at week 108, 79%, 59%, and 30% of patients achieved ASAS20, ASAS40, and ASAS5/6 response, respectively, and the BASDAI scores were improved at a percentage of 57%. Improvements in spinal mobility measures were sustained for all patients [30].

The study was extended even more, up to five years, by Martín-Mola et al. [31]. Thirty-seven patients completed the study and the results were satisfying. The improvements in all domains persisted and no new safety issues were reported [31].

Another randomized, placebo-controlled trial was carried out by Davis et al. [32] in order to assess the safety and efficacy of ETN in AS patients. One hundred and thirty-eight patients were treated with 25 mg ETN SC twice weekly and 139 patients were treated with placebo. At week 12, 59% of ETN patients achieved an ASAS20 response compared with 23% of placebo patients. At week 24, the corresponding percentages were 57% and 22% (p<0.0001). At week 24, partial remission was achieved by 17% of ETN patients and 4% of placebo patients. The BASDAI scores and spinal mobility measures also showed statistically significant improvements in ETN patients, compared to placebo patients, at weeks 12 and 24. The adverse events were similar in both groups, except three that were more frequent in the ETN group: injection-site reaction, upper respiratory tract infection, and accidental injury. In general, adverse events were mild and manageable [32].

Davis et al. [33,34] extended the study to an open-label up to 192 weeks. Placebo patients who started receiving ETN in the open-label phase exhibited a rapid improvement, as 70% of them achieved an ASAS20 response after 24 weeks. At week 96, 60% of the ETN/ETN group and 62% of the placebo/ETN group were ASAS40 responders, and 52% and 41%, respectively, were ASAS 5/6 responders. After week 96, 50 mg of ETN was administered SC once weekly. At week 192, 81% of patients were ASAS20 and 69% of them were ASAS40 responders. All improvements were maintained until week 192 without unexpected adverse events [33,34].

Brandt et al. [35] also examined the efficacy of ETN in a six-week randomized placebo-controlled trial, which was followed by an open-label phase. Fourteen patients received 25 mg ETN SC twice weekly and 16 patients received a placebo for six weeks. After that, all patients were treated with ETN. At week 6, the ASAS20 response was achieved by 78.6% of ETN group patients versus 25% of the placebo group patients, and the ASAS50 response was achieved by 42.9% versus 12.5% (p<0.01). BASDAI was improved from 6.5±1.2 to 3.5±1.9 for the ETN group while there was no improvement for the placebo group (p=0.003). BASMI and BASFI scores were also improved significantly in the ETN group. CRP levels had a remarkable reduction in the ETN group but not in the placebo group (p=0.001). At week 12, 71% of ETN/ETN group patients and 56% of the placebo/ETN group patients achieved BASDAI50 response. Ten out of 30 patients achieved partial remission at week 12. There were no withdrawals due to adverse events [35].

Baraliakos et al. [36,37] examined the outcomes of ETN treatment at two and seven years, by extending the previous study. Twenty-one of 30 patients and 16/30 patients completed the two-year and seven-year study, respectively. At week 102, BASDAI50 response was achieved by 53.8%, ASAS40 was achieved by 53.8%, and ASAS5/6 was achieved by 57.7% of patients. All measures of BASDAI, BASMI, and BASFI scores remained improved. Magnetic resonance imaging (MRI) assessment at two years showed a 75% amelioration in active spinal lesions. However, minor spinal inflammation persisted in 64% of patients. Evaluating all the results for up to seven years proved that ETN has durable efficacy and a good safety profile [36,37].

The collective findings from these studies (Table 3) consistently demonstrate that ETN is highly effective in treating AS. Across different durations and phases of treatment, ETN significantly improves key clinical measures such as ASAS response rates, BASDAI scores, spinal mobility, and inflammatory markers (CRP and ESR). The long-term extensions of the referenced studies indicate that these benefits are sustained over several years, suggesting that ETN remains effective and safe for chronic use. Adverse events are generally mild and manageable, with a safety profile comparable to a placebo in controlled trials. ETN provides substantial and sustained improvements in both clinical symptoms and quality of life. The consistent efficacy and safety across multiple trials and extended observation periods strengthen the case for ETN as a valuable choice in managing AS.

**Table 3:** Summary of studies for etanercept ETN, etanercept; IV, intravenously; ASAS, ASsessment in AS; ankylosing spondylitis; CRP, C-reactive protein; ESR, erythrocyte sedimentation rate; BASDAI, Bath AS Disease Activity Index; BASMI, Bath AS Metrology Index; BASFI, Bath AS Functional Index; MRI, magnetic resonance imaging.

Study	Patient groups/Dose	Frequency	Results
Calin et al., 2004 [29]	(a) ΕΤΝ 25 mg IV, (b) placebo	Twice per week	Week 12 (a) vs (b):
			ASAS 20: 60% vs 23%, ASAS 50: 50%, much more patients than group (b), ASAS 50: 25%, much more patients than group (b)
			(a): CRP↓ 70%, ESR↓ 80%
Dijkmans et al., 2009 [30]	Extension of the previous study as an open-label study, all patients received ETN after week 12	Twice per week	Week 108:
			ASAS 20: 79%, ASAS 40: 59%, ASAS 5/6: 30%
			BASDAI: 57% improvement
			Spinal mobility improvements were maintained
Martín-Mola et al., 2010 [31]	Re-extension of first study up to 5 years	Twice per week	Improvements were maintained for up to 5 years, with no drug safety issues
Davis et al., 2003 [32]	(a) ΕΤΝ 25 mg IV, (b) placebo	Twice per week	Week 12 (a) vs (b):
			ASAS 20: 59% vs 23%
			Week 24 (a) vs (b):
			ASAS 20: 57% vs 22%, ASAS partial remission: 17% vs 4%
Davis et al., 2005 [33], Davis et al., 2008 [34]	Extension of the previous study as an open-label study, all patients received ETN for up to 192 weeks. (a) ΕΤΝ/ETN, (b) placebo/ETN	Twice per week	Rapid improvement in patients who started receiving ETN after a placebo
			Week 96 (a) vs (b):
			ASAS 40: 60% vs 62%, ASAS 5/6: 52% vs 41%
			Week 192 total:
			ASAS 20: 81%, ASAS 40: 69%
Brandt et al., 2003 [35]	(a) ΕΤΝ 25 mg IV, (b) placebo. After week 6, all patients received ETN, (c) ETN/ETN, (d) placebo/ETN	Twice per week	Week 6 (a) vs (b):
			ASAS 20: 78.6% vs 25%, ASAS 50: 42.9% vs 12.5%
			BASDAI: significant improvement vs no improvement
			Week 12 (c) vs (d):
			BASDAI 50: 71% vs 56%
			ASAS partial remission: 10/30 patients in total
Baraliakos et al., 2005 [36], Baraliakos et al., 2013 [37]	Extension of previous study up to 7 years	Twice per week	Week 102:
			BASDAI 50: 53.8%
			ASAS 40: 53.8%, ASAS 5/6: 57.7%
			BASDAI, BASMI, BASFI: remained improved
			MRI (2 years):
			75% improvement in active spinal lesions
			Mild inflammation remained in 64% of patients

Golimumab

Reveille [38] examined the efficacy and safety of GLM in a one-year study. One hundred and five patients received GLM 2 mg/kg IV at weeks 0 and 4, and every eight weeks thereafter. One hundred and three patients received a placebo at weeks 0, 4, and 12, and then switched to GLM at weeks 16 and 20, and every eight weeks thereafter for 52 weeks. At week 16, 73.3% of GLM patients were ASAS20 responders, compared with 26.2% of placebo patients. Also, a clinically important improvement in ASDAS at week 16 was reported by 82.7% of GLM patients compared with 22.5% of placebo patients. At week 16, more GLM patients had ASAS40, ASAS5/6, partial remission, and BASDAI50 responses than placebo patients. ASDAS scores at weeks 28 and 52 in both groups were equivalent. In general, four weeks after placebo patients started receiving GLM (week 20), a rapid improvement was observed in placebo patients, and most scores were similar in both groups. The response was stable in both groups throughout the year. Furthermore, quality of life was assessed in both groups with SF-36 and ASQoL scores. Greater improvements in quality of life were observed in the GLM group patients at week 16. At week 28, and up to the end of the study, the improvements were similar and were sustained in both groups. A better BASDAI score was linked with a better quality of life. The most common adverse events were infections, and only four patients discontinued the study because of serious adverse events [38,39].

Inman et al. [40] assessed GLM’s efficacy in three groups. One hundred and thirty-eight patients received 50 mg of GLM SC, 140 received 100 mg of GLM SC, and 78 received a placebo. At week 12, the ASAS20 response was achieved by 59.4%, 60%, and 21.8% of patients in each group, respectively (p<0.001). At week 24, 43.5%, 54.3%, and 15.4% of patients in each group, respectively, achieved an ASAS40 response. Also, at week 24, 50.8%, 47.8%, and 14.7% of patients in each group, respectively, achieved BASDAI50 response. Clearly, there was a better treatment response in the GLM groups. Patients who received GLM showed improvements in BASFI, BASMI, SF-36 PCS, and SF-36 MCS scores, compared with the placebo patients. Nine patients discontinued the study because of adverse events, and in three of them increased liver transaminase levels were reported. In general, GLM was well tolerated [40].

Van der Heijde et al. [41] assessed the disease activity and quality of life in the previous three groups until two years. At weeks 14 and 24, major improvements in ASDAS scores were reported in 37%, 38.5%, and 5.1% (p<0.001) and in 39.4%, 39%, and 4.2% (p<0.001) of patients in each group, respectively. After week 16 (early escape) and week 24, all placebo patients were switched over to GLM. At weeks 52 and 104, improvements in ASDAS were similar in all three groups. Overall, patients who attained either major improvement in ASDAS or reached inactive disease status experienced a notably greater improvement in health-related quality of life (HRQoL), up to week 104 [41].

Bao et al. [42] examined GLM’s efficacy in Chinese patients. One group received placebo at weeks 0 to 20 and then received 50 mg of GLM SC at weeks 24 to 48, every four weeks. The other group received 50 mg of GLM SC until week 48, every four weeks. At week 14, ASAS20 response was achieved by 49.1% of GLM patients compared with 24.8% of placebo patients (p<0.001), and 50% vs 22.9%, respectively, at week 24 (p<0.001). At week 52, about 70% of all patients who received GLM had achieved an ASAS20 response. At week 14, mean improvements in BASFI score were -1.26 vs 0.11, p<0.001 and in BASMI score were -0.42 vs -0.19, p=0.021. More GLM than placebo patients achieved ASAS40 and ASAS 5/6 responses. GLM-treated patients also experienced improvements in HRQoL scores [42].

The collective findings from GLM studies (Table 4) consistently demonstrate the efficacy and safety of GLM in treating AS. Across different durations and patient populations, GLM significantly improves clinical measures such as ASAS response rates, BASDAI, BASFI, and BASMI scores, and reduces disease activity (ASDAS). Quality-of-life measures (SF-36 and ASQoL) also show notable improvements with GLM treatment. The safety profile is generally favorable, with most adverse events being mild and manageable. GLM seems to be an effective treatment option for AS, providing sustained improvements in both clinical symptoms and quality of life over long-term use. 

**Table 4:** Summary of studies for golimumab GLM, golimumab; IV, intravenously; ASAS, ASsessment in AS; AS, ankylosing spondylitis; ASDAS, Ankylosing Spondylitis Disease Activity Score; BASDAI, Bath AS Disease Activity Index; SF-36, Short Form-36, ASQoL, Ankylosing Spondylitis Quality of Life Questionnaire; BASFI, Bath AS Functional Index; BASMI, Bath AS Metrology Index; PCS, physical component summary score; MCS, mental component summary score; HRQoL, health-related quality of life.

Study	Patient groups/Dose	Frequency	Results
Reveille et al., 2019 [38], Reveille et al., 2020 [39]	(a) GLM 2 mg/kg IV, (b) placebo that changed in GLM	(a): Weeks: 0, 4, and then every 8 weeks. (b): Weeks: 0, 4, 12, and then change in GLM weeks: 16, 20, and then every 8 weeks	Week 16 (a) vs (b):
			ASAS 20: 73.3% vs 26.2%
			ASDAS (clinically significant improvement): 82.7% vs 22.5%
			More patients from group (a) compared to patients from group (b) achieved improvements: ASAS 40, ASAS 5/6, ASAS partial remission, BASDAI 50
			(a)>(b): improvements in quality of life (SF-36, ASQoL)
			After week 20:
			Rapid improvement of group (b) was observed, and most scores of the two groups were equalized
Inman et al., 2008 [40]	(a) GLM 50 mg SC, (b) GLM 100 mg SC, (c) placebo	Every 4 weeks	Week 12 (a) vs (b) vs (c):
			ASAS 20: 59.4% vs 60% vs 21.8%
			Week 24 (a) vs (b) vs (c):
			ASAS 40: 43.5% vs 54.3% vs 15.4%
			BASDAI 50: 50.8% vs 47.8% vs 14.7%
			Groups (a) and (b) showed significant improvements in scores: BASFI, BASMI, SF-36 PCS and SF-36 MCS, compared to group (c)
Van der Heijde et al., 2014 [41]	Evaluation of quality of life in the three groups of the previous study. After week 16 (early escape), and week 24, all patients received GLM	Every 4 weeks	Week 14 (a) vs (b) vs (c):
			ASDAS (significant improvement): 37% vs 38.5% vs 5.1%
			Week 24 (a) vs (b) vs (c):
			ASDAS (significant improvement): 39.4% vs 39% vs 4.2%
			Weeks 52 and 104:
			ASDAS improvements were similar in all three groups
Bao et al., 2014 [42]	(a) GLM 50 mg SC, (b) placebo that changed in GLM	(a): Every 4 weeks. (b): placebo for 20 weeks, then received GLM 50 mg SC every 4 weeks for a total period of 48 weeks	Week 14 (a) vs (b):
			ASAS 20: 49.1% vs 24.8%
			BASFI (average improvement): -1.26 vs -0.19
			BASMI (average improvement): -0.42 vs -0.19
			Week 24 (a) vs (b):
			ASAS 20: 50% vs 22.9%
			More patients receiving GLM than those receiving placebo achieved improvements in ASAS 40, ASAS 5/6, and improvements in quality of life (HRQoL)

Certolizumab pegol

Landewé et al., in 2014 [43], examined the efficacy of cerolizumab pegol in patients with active axial spondyloarthritis (axSpa), including non-radiographic axSpa (nr-axSpa) and AS. One hundred and eleven patients received 200 mg CZP SC every two weeks, 107 patients received 400 mg CZP SC every four weeks, and 107 patients received a placebo. The following results refer to the AS population of the study: ASAS20 response was achieved by 56.9%, 64.3%, and 36% of patients in each group, respectively, at week 12, and 67.7%, 69.6%, and 33.3% of patients in each group, respectively, at week 24. The percentages of ASAS40 responders were 40%, 50%, and 19.3% of patients in each group, respectively, at week 12, and 47.7%, 58.9%, and 15.8% of patients in each group, respectively, at week 24. The improvement in disease activity for patients in the CPZ groups was also shown by the changes in ASDAS scores: (-1.9, -1.7, and -0.6, respectively, at week 24). BASDAI, BASFI, and BASMI scores also suggested a greater improvement in the CPZ groups compared with the placebo group. Serious adverse events were developed by 4.7% of all CZP-treated patients and 4.7% of placebo-treated patients, but no deaths or malignancies were reported [43].

Sieper et al. [44] extended the study till week 96 and found that improvements in all scores (ASAS20, ASAS40, ASDAS, BASDAI, BASFI, and BASMI) were sustained until week 96, without any unexpected safety issues. They also noticed that patients with AS and nr-axSpa who received CZP reported greater improvements in nocturnal back pain, fatigue, ASQoL, SF-36 domains, and SF-36 PCS and MCS, compared with the patients who received a placebo [44,45].

Together, these findings (Table 5) underscore CZP's robust and sustained efficacy in reducing disease activity and improving QoL for patients with axSpa and AS, making it a valuable therapeutic option for the long-term management of these conditions.

**Table 5:** Summary of studies for certolizumab pegol CZP, certolizumab pegol; SC, subcutaneously; ASAS, ASsessment in AS; AS, ankylosing spondylitis; ASDAS, Ankylosing Spondylitis Disease Activity Score; BASDAI, Bath AS Disease Activity Index; BASFI, Bath AS Functional Index; BASMI, Bath AS Metrology Index; ASQoL, Ankylosing Spondylitis Quality of Life Questionnaire; SF-36, Short Form-36; PCS, physical component summary score; MCS, mental component summary score.

Study	Patient groups/Dose	Frequency	Results
Landewé, et al., 2014 [43]	(a) 200 mg CZP SC, (b) 400 mg CZP SC, (c) placebo	(a): Every 2 weeks. (b): Every 4 weeks. (c): Every 2 weeks	*The results refer to the study patients with ankylosing spondylitis
			Week 12 (a) vs (b) vs (c):
			ASAS 20: 56.9% vs 54.3% vs 36%, ASAS 40: 40% vs 50% vs 19.3%
			Week 24 (a) vs (b) vs (c):
			ASAS 20: 67.7% vs 69.6% vs 33.3%, ASAS 40: 47.7% vs 58.9% vs 15.8%
			ASDAS (change): -1.9 vs -1.7 vs -0.6
			BASDAI, BASFI, and BASMI had greater improvement in the CZP-treated groups than in the placebo-treated group
Sieper et al., 2015 [44,45]	Extension of the previous study for 96 weeks		Greater improvements for patients receiving CZP compared to those receiving placebo in the following: nocturnal back pain, fatigue, ASQoL, SF-36, and SF-36 PCS and MCS
			Improvements for CZP-treated patients were maintained through week 96 for all scores: ASAS20, ASAS40, ASDAS, BASDAI, BASFI, and BASMI

## Conclusions

The more extensive use of TNF-α inhibitors represents a pivotal advancement in the management of AS, offering significant benefits in alleviating symptoms and improving patient outcomes. Through this literature review, it is evident that TNF-α inhibitors not only effectively mitigate inflammation but also contribute to the mitigation of structural damage as AS progresses. Furthermore, the observed improvements in disease activity, patient functioning, and patient QoL underscore the clinical relevance of TNF-α inhibitors in this exhausting condition. However, ongoing research is warranted to explore long-term efficacy, safety profiles, and potential combination therapies to optimize treatment strategies for AS patients. Overall, TNF-α inhibitors now stand as a cornerstone in the management of AS, offering hope for improved disease control and enhanced QoL for affected individuals.
